# Supplementary material for: Mammalian Models in Alzheimer’s Research: An Update
Source: Cells. 2023 Oct 16;12(20):2459. doi: 10.3390/cells12202459 (PMC10605533; doi:10.3390/cells12202459)
Supplement: Supplementary file 1 [file cells-12-02459-s001.zip › cells-2619619-supplementary.pdf]

Supplementary Table S1: Transgenic mice models of Amyloid Pathology

Model	Mutation	Model Background	Aβ Pathology	Disadvantages	References
**Tg2576**	APP KM670/671NL (Swedish)	C57BL/6 x SJL	Plaques by 11-13 months	Overexpression of multiple APP fragments<br>Random integration of transgenes<br>Sudden death may occur (Tg2576)<br>Aβ accumulation is preceded by cognitive impairment<br>Genetic backgrounds mixed in some cases	27, 33-35, 168
**PDAPP**	APP V717F (Indiana)	C57BL6 x DBA2	Plaques at 6 months		27,28,29,31,168
**J20**	APP K670_M671delinsNL (Swedish), APP V717F (Indiana)	C57BL/6	Plaques by 5-7 months		26, 45-48,168
**TgCRND8**	APP K670_M671delinsNL (Swedish), APP V717F (Indiana)	Hybrid C3H/He-C57BL/6	Plaques by 3 months		49-52, 168

**5xFAD (B6SJL)**	APP KM670/671NL (Swedish), APP I716V (Florida), APP V717I (London), PSEN1 M146L, PSEN1 L286V	C57BL/6 x SJL	Plaques by 1.5 months	Overexpression of multiple APP fragments<br>No perfect negative control<br>Multiple mutations (APP+PS1)<br>Occurrence of non-specific ER stress<br>No NFTs<br>Cross breeding may be complicated in some cases<br>Random integration of transgenes	26,27,168
**5xFAD (C57BL6)**	APP KM670/671NL (Swedish), APP I716V (Florida), APP V717I (London), PSEN1 M146L, PSEN1 L286V	C57BL6	Plaques by 1.5-2 months		59,60,62,58
**APPxPS1**	APP K670_M671delinsNL (Swedish) and PSEN1 L166P	C57BL/6	Plaques by 1.5 months (cortex) and 3-4 months (hippocampus)		63-67
**TREM2- BAC x 5xFAD**	APP KM670/671NL (Swedish), APP I716V (Florida), APP V717I (London), PSEN1 M146L, PSEN1 L286V	TREM2-BAC: FVB/NJ;<br>5xFAD: C57BL/6 x SJL	Plaques by 8.5 months	No NFT or neuronal loss	26,69-72
**AppNL-F**	APP KM670/671NL (Swedish), APP I716F (Iberian)	C57BL/6	Plaques by 6 months	No NFT or neuronal loss<br>Multiple FAD mutations of APP<br>Overproduction of CTF-β<br>Genomic homozygosity	26,68,168
**APPV717I**	APP V717I (London)	C57BL/6 x FVB/N	Plaques by 10 months	No NFT observed	43,44

**Tg-SwDI**	APP K670_M671delinsNL (Swedish), APP E693Q (Dutch), APP D694N (Iowa)	C57BL/6	Plaques by 3 months	No NFT observed	26,53,54

Abbreviations: Aβ (amyloid-beta); NFT (Neurofibrillary Tangles); APP (Amyloid Precursor Protein); FAD (familial Alzheimer's Disease); CTF-β (C-terminal fragment beta); ER (Endoplasmic Reticulum)

Supplementary Table S2: Transgenic mice model of Tau pathology

Model	Mutation	Model Background	Tau Pathology	Disadvantages	References
**JNPL3**	MAPT P301L	C57BL/6, DBA/2, SW mixed background	NFTs develop from 4.5-6.5 months	Absence of amyloid pathology	82-85
**hTau**	M.A.P.T.: knock out; M.A.P.T.: Transgenic	Cross between SW and B6D2F1	Hyperphosphorylated tau at 6 months	Absence of amyloid pathology	88-90,232
**rTg (tau P301L)**	MAPT P301L	Produced by crossing 129S6 (activator) to F.V.B. (responder)	Pretangles at 2.5 months. Argyrophilic tangle-like inclusions in the cortex by 4 months and in the hippocampus by 5.5 months	No association found between genetic mutation and tau pathology in AD pateints<br>Overexpression of mutant human tau may attribute to	26,88,89,97,232

				behavioral phenotypes and neuropathological deficits.<br>No amyloid pathology	
**PS19 (tau P301S)-**	MAPT P301S	(C57BL/6 x C3H)F1	NFTs develop at 6 months	No association found between genetic mutation and tau pathology in AD pateints<br>No amyloid pathology	26,100,232

Abbreviations: NFT (Neurofibrillary Tangles); MAPT (Microtubule Associated Protein Tau); AD (Alzheimer's Disease)
